# A Perspective on Implementation of Technology-Driven Exergames for Adults as Telerehabilitation Services

**DOI:** 10.3389/fpsyg.2022.840863

**Published:** 2022-03-17

**Authors:** Cécil J. W. Meulenberg, Eling D. de Bruin, Uros Marusic

**Affiliations:** ^1^Institute for Kinesiology Research, Science and Research Centre of Koper, Koper, Slovenia; ^2^Department of Health Sciences and Technology, Institute of Human Movement Sciences and Sport, Zurich, Switzerland; ^3^Division of Physiotherapy, Department of Neurobiology, Care Sciences and Society, Karolinska Institutet, Solna, Sweden; ^4^Department of Health, OST – University of Applied Sciences of Eastern Switzerland, St. Gallen, Switzerland; ^5^Alma Mater Europaea – ECM, Department of Health Sciences, Maribor, Slovenia

**Keywords:** exergames, functionality, mental health, dual-task, telehealth, tele rehabilitation

## Abstract

A major concern of public health authorities is to also encourage adults to be exposed to enriched environments (sensory and cognitive-motor activity) during the pandemic lockdown, as was recently the case worldwide during the COVID-19 outbreak. Games for adults that require physical activity, known as exergames, offer opportunities here. In particular, the output of the gaming industry nowadays offers computer games with extended reality (XR) which combines real and virtual environments and refers to human-machine interactions generated by computers and wearable technologies. For example, playing the game in front of a computer screen while standing or walking on a force plate or treadmill allows the user to react to certain infrastructural changes and obstacles within the virtual environment. Recent developments, optimization, and minimizations in wearable technology have produced wireless headsets and sensors that allow for unrestricted whole-body movement. This makes the virtual experience more immersive and provides the opportunity for greater engagement than traditional exercise. Currently, XR serves as an umbrella term for current immersive technologies as well as future realities that enhance the experience with features that produce new controllable environments. Overall, these technology-enhanced exergames challenge the adult user and modify the experience by increasing sensory stimulation and creating an environment where virtual and real elements interact. As a therapy, exergames can potentially create new environments and visualizations that may be more ecologically valid and thus simulate real activities of daily living that can be trained. Furthermore, by adding telemedicine features to the exergame, progress over time can be closely monitored and feedback provided, offering future opportunities for cognitive-motor assessment. To more optimally serve and challenge adults both physically and cognitively over time in future lockdowns, there is a need to provide long-term remote training and feedback. Particularly related to activities of daily living that create opportunities for effective and lasting rehabilitation for elderly and sufferers from chronic non-communicable diseases (CNDs). The aim of the current review is to envision the remote training and monitoring of physical and cognitive aspects for adults with limited mobility (due to disability, disease, or age), through the implementation of concurrent telehealth and exergame features using XR and wireless sensor technologies.

## Introduction

During in-person health care, rehabilitation outcomes after injury in conjunction with disease, are dependent on the adherence of the patient to the therapy. Time spent in therapy and high commitment to the intensity of the exercises, being crucial factors in recovery (e.g., Borghese et al., 2014; Piech and Czernicki, 2021). The proper evaluation of such face-to-face in-person therapies for non-communicable cognitive-motor diseases and their specific constructs, can boost recovery and prevent the worsening of disease symptoms. Especially, implementation of real-life activities and cognition structurally assessed over time.

During the worldwide COVID-19 outbreak of the last 2 years, the implementation of restrictions in health care (lockdowns with travel restrictions and social distancing), overwhelmingly diminished personal contact between care giver and patients (Mantovani et al., 2020; Singh et al., 2020; Ong et al., 2021). This most severely affected mobility-impaired elderly and sufferers from CNDs due to the stagnation in on-going immediate care. Thus public health care authorities were confronted with two major challenges: (1) how to maintain in-person health services including personal rehabilitation therapies for mobility-impaired elderly; (2) how to encourage (older) adults to remain both physically and cognitively active.

Trying to overcome the most obvious pandemic restrictions, already authorities retreated to several forms of telecommunication health care (Mantovani et al., 2020; Singh et al., 2020; Ong et al., 2021). Even though it is not widely implemented nor developed. Such telehealth could potentially provide several formats of clinical and therapeutic services at a distance. This is especially needed since the effects of the restrictions and the consequently neglected health care, in combination with enlarged waiting lists, are already showing major financial concerns. Besides, on top of that, will push a demand for both cognitive and physical health services in the near and far future (Mantovani et al., 2020; Singh et al., 2020).

The current review will set forth the properties required to implement successful telerehabilitation from the point of adherence and effectiveness in physical and cognitive rehabilitation. It will further describe the existing virtual and wireless technologies to remotely assess physical and cognitive health. Finally we give examples of some recently developed exergames for (older) adults.

## Search Strategy and Study Selection

Scientific literature in English language was acquired through searches conducted on PubMed/MEDLINE (NLM), database until March 1, 2021. A search for manuscripts on “exergame” and “serious game” in combination with “cognitive-motor,” “dual-task” and “biomarker,” “virtual reality” (with specific deviations of keyword combinations, such as “functionality,” “motor intervention,” “physical activity,” “cognition,” “training,” “mental health,” “augmented reality,” “mixed reality,” etc.) was conducted. Furthermore, these selections were then refined to compile recent manuscripts (preferably publication date <5 years) of interest regarding telerehabilitation games dealing with specific clinical patients like dementia and cognitive impairment, chronic obstructive pulmonary disease (COPD), post-stroke, multiple sclerosis (MD), and Parkinson’s disease (PD). Additional searches were focused on telerehabilitation requirements, remote and wireless technologies that could potentially support the architecture of distant health care at home. Finally, some recent perspective papers on virtual reality and exergames in relation to the COVID-19 pandemic were included.

## General Traditional Rehabilitation

Injured older adults and patients with CNDs that are somehow mobility-impaired, are well-served by proper therapy for the recovery of physical and cognitive abilities. Patients after stroke, various dementias, and other neurodegenerative disorders. Walking, balance, posture, and also assistance in fall prevention, next to the transfer of these abilities to everyday life activities – if (re)learned well – are most important for improving quality of life. To achieve such improvement, the outcomes and the success of the therapies depend on the appropriate intervention duration and repetition. With therapy difficulties and challenges that need a personalized complexity depending on the various patients (e.g., Borghese et al., 2014; Piech and Czernicki, 2021). In the *status quo* of the service models of rehabilitation, (in- and out-)patients are primarily treated in one-on-one sessions. Prolonged post-acute rehabilitation is often missing. Thus, threatening full recovery. The number of in-person contact hours is an important parameter determining rehabilitation success. However, these are also crucial to achieve both highly effective therapy outcomes and high adherence in the long-term. The recent pandemic strongly supports the need for remotely (health server) controlled, digital, home-based training. Telerehabilitation approaches and digital/technological solutions are promising options to overcome barriers of accessibility, discontinuity, and lack of resources.

## Technology-Driven Exergames

Exergames that require adult participants to partake in game-like, technology-driven physical exercise have been offering opportunities in enhancing adherence through a playful way (e.g., Gavelin et al., 2021; Yen and Chiu, 2021). During physical rehabilitation, often, the in-person therapy with exergames, includes the use of pressure sensitive plates or treadmills that are positioned in front of a computer screen (Zucchella et al., 2014). The software is enabled to project virtual reality (VR) environments with infrastructural changes and obstacles, to which the participants need to react (Piech and Czernicki, 2021). Overall, these achieve greater presence and engagement than standard rehabilitation setups (see Zucchella et al., 2014; Gavelin et al., 2021; Yen and Chiu, 2021; for recent overviews), like laboratory treadmill/force plate exercises.

Virtual reality can be understood as an environment that is created by a computer or other media and in which the user has a feeling of being present in the environment (Biocca, 1992). The technological change that took place in the video game sector showed a development from conventional two-dimensional (2D) virtual environment use towards playing video games in a stereoscopic three-dimensional (3D) environment. For example, cubes and other 360°-projection setups, or by using a head-mounted-display (Roettl and Terlutter, 2018). Such wearable and wireless VR headsets enable non-restricted whole-body movements and enhance the degrees of functionality while playing the game. Thus complementing the VR immersiveness.

The recent field of mobile body/brain imaging (MoBI, e.g., Gennaro and de Bruin, 2018; Greeley et al., 2021) contributes wireless sensors that register simultaneous whole-body motion and brain activity (Berger et al., 2019). For example through electroencephalography (EEG) and infrared scanning. MoBI will facilitate our understanding of brain and body dynamics. Both under more naturalistic circumstances and under laboratory conditions assessing real-life physical activities, like walking and certain cognitive domains. Besides, MoBI in combination with remotely controlled XR environments, certainly will enhance the user’s sensory stimulation.

While technology-enhanced exergames for adults incorporating XR and MoBI are slowly becoming a reality (Singh et al., 2020; Ong et al., 2021; Piech and Czernicki, 2021; Yen and Chiu, 2021, see below and in the Supplementary Table), they provide safe laboratory conditions for training of real-life activities that are investigator-controllable. From the perspective of the participant, XR-MoBI challenges and enhances autonomy, curiosity, motivation and increases adherence (Gennaro and de Bruin, 2018). The participant’s reaction to the exercises becomes a physically active challenge under more naturally relaxing circumstances, providing potentially better conditions to assess a participant’s cognitive health safely (e.g., Bernini et al., 2021). Furthermore, XR-MoBI provides more relevant natural biomarkers of physical activity, cognition and especially real-life activities. Thus, ultimately achieving greater engagement than standard exercise. In the case of CNDs and neuro-motor disorders, real-life activities are only sparsely being monitored and trained correctly within exergames. Even so, lower limb gait parameters are still fairly neglected. This becomes a reality in monitoring and assessing through XR-MoBI. On the other hand, wireless and wearable sensors require adjustment and training of the adult to get used to. But these circumstances can be conditioned, and in general are experienced as less irritable than wired laboratory conditions and desk tests. Consequently, it seems reasonable to extrapolate the wish for such combined XR-MoBI setups to implement under home and remotely controlled user-conditions.

## Telehealth Architecture

Although there is a high demand for rehabilitative treatment, health care systems often fail to provide sufficient resources (e.g., qualified persons, time, and money). Especially, because of being restricted to in- and out-patient rehabilitation that mainly depending on face-to-face treatment. Furthermore, most existing health care services rather target the acute care without considering the necessities of continuous treatment and centralized case management. In many European countries this leads to a restriction of extensive therapies for in- or out-patient rehabilitation to a too short time (e.g., 3–4 weeks). Notwithstanding the knowledge that continuous training and support for a prolonged rehabilitation time is important to ensure maximal recovery. Geriatric patients for example often leave the rehabilitation clinic without reaching their full recovery potential (Tillou et al., 2014). Consequently, a health care system should be developed that provides integrated hardware and software solutions for rehabilitation, covering the whole continuum-of-care. Interestingly, the potential incorporation of adult exergames and XR-MoBI technologies into telehealth, comes at a time that society is having a demand for such exergames due to the COVID-19 pandemic. As it is that under lockdown conditions health care obviously becomes limited due to general measures (Mantovani et al., 2020; Singh et al., 2020; Ong et al., 2021; Piech and Czernicki, 2021; Stasolla et al., 2021). Indeed the COVID-19 pandemic actually boosted the number of telehealth trials, with increasing efforts of health care performed remotely via telecommunication technology. Although most of the telehealth with exergames was performed unprepared and as a pilot action, its reception shows promising results from the perspective of both care giver and patient (e.g., Bernini et al., 2021). In general the telerehabilitation opportunity seems to have attracted attention of ICT developers, health care units, insurance companies and patient associations.

Telehealth is a fairly new discipline and its distant character, seems to be fairly well suited to incorporate the new developments in exergames for adults with the XR-MoBI technologies. Especially, the feature of a non-physical contact between care giver and patients. Telerehabilitation and exergames, like the latest gaming sector output, embrace general features like home-setups, wearable technology and internet access. However, telerehabilitation, just like in-person doctor’s consult requires: intake, prescription, monitoring, adaptation and feedback. Hence, a successful telerehabilitation architecture would require a similar structure. Obviously both a hospital/care unit and a patient (home) unit are needed (Borghese et al., 2014; Bernini et al., 2021). While for larger adherence and social acceptance by patients, it is suggested to include a networking/community unit (Borghese et al., 2014; Stasolla et al., 2021). This enables accessibility to both patient and care giver, facilitates that patients can meet and share experiences, and provides that feedback merges and becomes treatment.

While general traditional rehabilitation requires an appropriate intervention duration, repetition, personalized complexity and patient-specific difficulties and challenges, so does the telerehabilitation intervention. Potential exergames also require attention for personalization, task variability, progression, and continuous adaptation of task difficulty, in order to increase the patient’s skills and therapeutic outcomes (e.g., Borghese et al., 2014; Piech and Czernicki, 2021). Thus, successful telerehabilitation architecture requires a certain consistency in the way therapeutic protocols are delivered, performance recorded, feedback is provided and progress is monitored. Preferably through separate units.

## Exergames for Adults That Assess Physical Activity and Cognition

Recently VR exergames for adults that monitor and train physical activity have been developed for: posture (Solis-Escalante et al., 2019; Carr et al., 2020; Imaoka et al., 2020), falls (Chen et al., 2020; Liston et al., 2021), dual-task walking (Kizony et al., 2017; Janouch et al., 2018; Kafri et al., 2021). As well as assessing cognitive health and providing comfort in patients with neurological diseases and communication difficulties (e.g., Stasolla et al., 2021). In addition, various exergames investigate the correlation of multisensory stimulation and age on realistic daily-life activities (Wechsler et al., 2018; Souza-Silva et al., 2019; Carr et al., 2020; Imaoka et al., 2020). Some also investigate the maintenance of posture and the response to fall and how they are age-dependently influenced by sound and vision (e.g., Stapleton et al., 2014; Lupo and Barnett-Cowan, 2018).

A dynamic posturography system for investigating balance while standing on a movable platform, combines with EEG according to MoBI principles. For various exercises, the dynamic adjustments in cortical brain activity that occurred were shown to be predictive of determinated posture and fall-prone behavior (Solis-Escalante et al., 2019). The applications of combined cortical EEG recordings (Jiang et al., 2021) and specific gait parameters (Pieruccini-Faria et al., 2021) can independently predict the development of cognitive impairment in older and cognitive impaired persons. However, the integration of such MoBI applications into exergames is limited, but will be elaborated on (Helbing et al., 2020). At the same time, XR-MoBI exergames with the appropriate unit-architecture of telerehabilitation are limitly published.

Exergames that train various cognitive domains are numerous, albeit under variable mobile circumstances. For example, while sitting with a tablet cognition can be trained, and a test battery can assess cognitive control (Boujut et al., 2020). On the other hand cognitive impairment causes variation in the magnitude of cognitive-motor integration, which in turn causes variation in posture and spatial aspects of gait (Mahoney and Verghese, 2020). Hence, several exergames successfully assessed cognitive memory domains while walking and performing realistic daily-life activities within VR environments (Kizony et al., 2017; Janouch et al., 2018; Kafri et al., 2021).

A computer-aided rehabilitation exercise simulation software shows potential to detect early and mild cognitive decline remotely (Bernini et al., 2021). The detection of associated poorer postural and listening task performances compared healthy and cognitively declined participants. It can early-on detect poorer global cognitive performance (Carr et al., 2019). An index extracted from measurements during VR in combination with a treadmill, motion sensors and a camera, can distinguish patients with Alzheimer’s disease, amnestic mild cognitive impairment and healthy adults (Tarnanas et al., 2013). The index correlates strongly with standard cognitive and physical activity measurements. Changes in postural sway by older adults are also indicative of cognitive impairment, and several VR exergames have been addressing this issue (Carr et al., 2019; Liao et al., 2019; Imaoka et al., 2020). Interestingly, 12 weeks training significantly improved dual-task gait performance in older mildly cognitively impaired adults (Liao et al., 2019). A spatial navigation VR platform is similarly feasible of detecting pre-dementia symptoms (Ijaz et al., 2019). A new participatory virtual platform is intended to be used as a rehabiliation gaming tool (Ferreira-Brito et al., 2020).

## The Application of Exergames in Other Chronic Non-Communicable Diseases

It also should be noted that recent research indicates that particular implemented VR exergames can yield biomarkers for early detection of various CNDs (e.g., Imaoka et al., 2020; Jiang et al., 2021; Pieruccini-Faria et al., 2021). These have the potential to be developed and integrated into rehabilitation programs.

For example, exergames with physical exercises tailored to the patient’s needs and abilities for older COPD patients (LeGear et al., 2016; Liu et al., 2016; Frade et al., 2019; Sutanto et al., 2019). A 3D motion analysis system with an instrumented treadmill and a VR 180 degrees projection screen (Liu et al., 2016), assessed the walking of COPD patients. It showed to be reproducible and valid for evaluating the physical activity capacity of adults with COPD (Liu et al., 2016; Frade et al., 2019), and even of healthy older persons (Liu et al., 2016).

Rehabiliation for post-stroke adults has been studied using simulated and treadmill dual-task walking exergames (Al-Yahya et al., 2016; Liu et al., 2017). A recent systematic review fails to show additional benefits of the implemented VR (Laver et al., 2017). Nevertheless recent studies suggest that different types of dual-task training should be adopted to enhance gait performance in stroke patients (Borghese et al., 2014; Liu et al., 2017; Vallejo et al., 2020). A recently developed exergames did provide autonomous home exercise, balance and gait training with high adherence for stroke patients (Held et al., 2018).

The disability of MS patients is strongly correlated with their mental tracking rate and dual-task performance. This has been tested during simultaneous walking and memory tests in both treadmill experiments and imagined virtual tasks (Downer et al., 2016). Combined with EEG recordings a positive correlation between load-related EEG effects and dual-task performance was established (De Sanctis et al., 2020). It was concluded that EEG can provide biomarkers of MS cognitive-motor dysfunction. An on-going study (Hsieh et al., 2020) aims to establish the benefits of a combined cognitive-motor VR training on MS symptoms, and compare the results to conventional treadmill training.

Parkinson’s disease patients seated in front of a laptop while using a markerless motion sensing training program for the forearms, wrists and hands (Van Beek et al., 2019), significantly improved the impaired dexterity of PD participants assessed at baseline. Besides, showed a high adherence and increased motivation. Cognition of PD patients was safely and effectively assessed by a patient-tailored home-rehabilitation simulation program (Bernini et al., 2021). In addition, PD patients performing cognitive-motor tasks have general benefits. A recent review without the focus on the use of VR (Pieruccini-Faria et al., 2021), mentioned that weak but significant associations exist between parkinsonian signs and gait variability. Besides, that gait variability is a better motor marker of cognitive performance than severity of parkinsonism. Under VR dual-task conditions across different cognitive function domains, this was further supported by Penko et al. (2018). They noted that diminished gait performance indicated a global PD-related deficit in information processing and regulation of gait.

## Telerehabilitation Exergame User and Expert Evaluations

The quantitative and qualitative assessment of exergames for adults that incorporate telerehabilitation architecture or show good promise to be in due time constructed according to the outlined requirements above, are only limited reported on. Here we summarize some of the user and expert evaluations (Table 1).

**TABLE 1:** Overview of available user and expert evaluations for selected telerehabilitation exergames.

Study	Description (*name*, setup, brief protocol)	Adult participants (average age, number of: participants/patients, males)	Evaluation
Bernini et al. (2021)	*HomeCoRe: Home Cognitive Rehabilitation* software, a computer-supported cognitive training program, patient-tailored intervention aimed at stimulating several cognitive abilities through a series of 2D exercises. An overall weighted score index takes into account the correctness of the answers, the execution time, and the difficulty of the exercises	Adults patients with early and mild cognitive impairment, and Parkinson’s disease patients	Patients and caregivers provided positive evaluation
Chen et al. (2020)	*Kinect 2.0*-incorporated system to capture and generate 3D models of the elderly and immerse them in an interactive virtual environment through screen projection	Healthy old (71.5 years, *N* = 25, 16 males)	The User Experience Questionnaire (UEQ-S) to assess user experience with averages for pragmatic quality score (1.652); hedonic quality score (1.880); overall score (1.776): rated good
Ferreira-Brito et al. (2020)	*NeuroVRehab.PT*: Image-based fully navigable and interactive virtual supermarket with navigation of medium-sized supermarket, and the use of shopping lists to memorize	Healthy old (70.92 years, *N* = 110, 28 males)	Through interviews with health professionals (*N* = 7) the application was assessed for the platform’s rehabilitation potential, clinical applicability and user experience Support that application is an ecologically valid platform with clinical applicability in neuro rehabilitation
Held et al. (2018)	*REWIRE-system*: Telerehabilitation for balance and gait training	First-time stroke patients with a mild to moderate residual deficit of lower extremities (56 years, *N* = 16, 9 males)	Patient survey through Technology Acceptance Model questionnaire after the training: excellent acceptance Patients were satisfied with and motivated in using the system
Liston et al. (2021)	*HOLOBalance*: Body-worn sensors (pressure detecting insoles, inertial measurement unit and a heart rate monitor) a head mounted augmented reality display and a depth camera	Old at risk for falls (*N* = 120), randomized to receive an 8-week home exercise program	Upcoming: The determine of safety, acceptability and feasibility of HOLOBalance
Van Beek et al. (2019)	*Leap Motion Controller*: Dexterity intervention with laptop, markerless motion sensing system that tracks the motion of both forearms, wrists, and hands	Parkinson’s disease patients (65.4 years, *N* = 10, 3 males)	High adherence and increased motivation The System Usability Scale was performed to assess the usability of application: rated acceptable to very good

An augmented reality (AR)-based exergame that intents to reduce fall risk, using kinect capturing and generating patients’ movement in VR, was evaluated for good user experience by older adults (Chen et al., 2020). *REWIRE* system, another telerehabilitation home system for balance and gait training (Held et al., 2018), was assessed by older stroke patients through the Technology Acceptance Model questionnaire. Excellent values of user satisfaction and motivation after the training were reported. Also including telerehabilitation architecture, is *HOLOBalance* that includes body-worn sensors and a head mounted AR-display with depth camera (Liston et al., 2021). In the future the authors will collect quantitative and qualitative data to explore the acceptability of providing holographic balance training programs to older adults at risk for falls.

*Leap Motion Controller* is a dexterity intervention with laptop and markerless motion sensing system that tracks the motion of both forearms, wrists, and hands (Van Beek et al., 2019). Ten Parkinson’s patients showed high adherence and increased motivation, besides evaluated the usability acceptable to very good.

Telerehabilitation exergames assessing cognition are also showing promise. *NeuroVRehab.PT* is an image-based navigable and interactive virtual supermarket in which shopping lists need to be memorized (Ferreira-Brito et al., 2020). Older male persons using *NeuroVRehab.PT* were assessed by health professionals, and they concluded the system is an ecologically valid instrument with clinical applicability in mild cognitive impairment neurorehabilitation. Patients with early and mild cognitive impairment and Parkinson’s disease patients used *Home Cognitive Rehabilitation* (*HomeCoRe*)-software, a computer-supported cognitive training program (Bernini et al., 2021). The software is in use in both homes and hospitals, and received positive evaluations from both patients and caregivers.

These evaluations of successful exergames for adults with telerehabilitation architecture show that indeed a diversity of patients can be served, even remotely, and that both patients and expert health practitioners do give positive user evaluations, and thus endorse clinical application. However, many assessment results, most likely, have not been published yet.

## Discussion

During pandemic lockdowns the societal measures applied will limit the regular contact of many patients and their care providers. To maintain certain levels of quality of life, many mobility-impaired patients might be assisted by telerehabilitation exergames for adults. These can both provide physical and cognitive exercises, mimicking real-life activities in an enriched environment. It is recommended to construct such exergames including an appropriate telerehabilitation structure with units for care giver, patients and its whole community. Such architecture will facilitate the planning of controllable exercises, with personalized variety, and should give the patient opportunity to leave feedback upon which can be acted by the health provider. It is envisioned that architectural control and feedback units for patient and caregiver will be able to complement, and partly replace, the need to meet in-person health care. At least for some aspects of the CNDs and neuro-motor disorders. However, only limited telerehabilitation exergames have currently seen such implemented structure.

In the future, the offered remote therapy, might be envisioned to include more aspects of technologies like XR in combination with wireless, wearable sensors and headsets. The more realistic environments offered will certainly facilitate the immersiveness and adherence of the patient. Besides, achieve greater presence with more freedom of movement and engagement than standard rehabilitation programs. Exergames that quantify body postures, i.e., sitting, standing, and walking with advancing age as exercises of daily-life are already plentiful available. And these can be used as telerehabilitation tool to regain postural control, maintenance of balance, and to improve motor abilities in general. In addition, the technology of more realistic brain and body dynamics (i.e., MoBI) has potential to contribute as well. But is limitedly applied (as of yet). Several exergames identified and established new biomarkers (for various CNDs) as indicative of therapy progress. In contrast, dual-task cognitive motor assessment and specifically lower limb tracking functions as early detection of various CNDs, are sparsely at hand as exergames. Certainly, many exergames are being developed in controlled laboratories, often as expensive elaborate demo units. These need to be scaled-down to being user-friendly, and require longitudinal testing on mobility-impaired adults.

The general perspective for adult exergames with implemented XR-MoBI technology is such that within the next years telerehabilitation will embrace more upgraded exergames, able to remotely perform long-distance assessments of overall locomotion, as well as cognitive health. The patient will be empowered through this therapeutic entertainment with enlarged adherence, equal to or larger than in-person rehabilitation. While health providers will embrace the exergames as new tools to provide additional and efficient health services.

## Data Availability Statement

The original contributions presented in the study are included in the Supplementary Material, further inquiries can be directed to the corresponding author/s.

## Author Contributions

CM, EB, and UM designed the research, performed the literature data extraction, drafted the manuscript, and edited and revised the manuscript. All authors contributed to the article and approved the submitted version.

## Conflict of Interest

The authors declare that the research was conducted in the absence of any commercial or financial relationships that could be construed as a potential conflict of interest.

## Publisher’s Note

All claims expressed in this article are solely those of the authors and do not necessarily represent those of their affiliated organizations, or those of the publisher, the editors and the reviewers. Any product that may be evaluated in this article, or claim that may be made by its manufacturer, is not guaranteed or endorsed by the publisher.
