# Supplementary material for: A Perspective on Implementation of Technology-Driven Exergames for Adults as Telerehabilitation Services
Source: Front Psychol. 2022 Mar 17;13:840863. doi: 10.3389/fpsyg.2022.840863 (PMC8968106; doi:10.3389/fpsyg.2022.840863)
Supplement: Supplementary file 1 [file Table_1.docx]

Supplementary Table 1 Overview of exergame studies that assess physical and cognitive activities in mobility-impaired adults.

| **Study** | **Description**  (*name*, setup, brief protocol) | **Adult participants** (average age, number of: participants / **patients**, males) | **Outcomes**  (general)  (***user and expert application evaluation***) | **Presence of telehealth architecture** (yes/no)  **Potential to upgrade to user-friendly** (yes/no)  **Requires down-scaling to home use** (yes/no) |
| --- | --- | --- | --- | --- |
| Al-Yahya et al., (2016) | Dual-task treadmill walking with cognitive task: prefrontal cortex activation measured with near-infrared spectroscopy, spatiotemporal gait parameters assessed with accelerometer, gyroscope, and magnetometer. | Healthy controls (54.4 years, *N* = 20, 12 males).  **Chronic stroke patients** (59.6 years, *N* = 19, 17 males). | The dual-task related increase in cortex activity correlated with dual-task-related change in behavior in stroke participants. Enhanced brain activity changes relate to dual-task motor decrements. | No  Yes  Yes |
| Bernini et al., (2021) | *HomeCoRe: Home Cognitive Rehabilitation* software, a computer-supported cognitive training program, patient-tailored intervention aimed at stimulating several cognitive abilities through a series of 2D exercises. An overall weighted score index takes into account the correctness of the answers, the execution time, and the difficulty of the exercises. | Applied to **patients with early and mild cognitive impairment, and Parkinson disease patients** | Applicable to patients with early and mild cognitive impairment.  ***Patients and caregivers provided positive evaluation.***  Already home simulations assessing functionality and usability through controlled in-hospital sessions. | Yes  Already done  No |
| Borghese et al., (2014) | *REWIRE*: Monitoring patient day-to-day lifestyle through worn sensors, while in addition activity signals upon *Intelligent Game Engine for Rehabilitation* instructed movements are recovered from depth and color imagery captured by *Kinect* 3D motion monitor. | **Stroke patients** | *REWIRE*-project shows the development of exergames with a telehabilitation structure consisting out of a hospital station, a networking station and a patient station, and provide data mining functionalities to discover patient's rehabilitation patterns. | Yes  Already done  No |
| Boujut et al., (2020) | *Neuropeak web platform*: Attentional control training in older people of updating (N-back type exercises), inhibition (Stroop-like exercises) computerized training or active control (general knowledge quiz game), working memory transfer tasks. | Healthy young (20-35 years; *N* = 30).  Healthy old (60-85 years, *N* = 90). | Attentional control training is effective in improving updating and inhibition performance on training tasks. The optimal dose to achieve efficacy is ∼9 half-hour sessions and the dose effect was related to difficulty level. | Yes  Yes  No |
| Carr et al., (2019) | *StreetLab*: VR street scene, curved projection screen combined with surround sound loudspeakers, and strain gage force plate. Battery of cognitive tests. | Healthy old with normal cognition (66.4 years; *N* = 14, 5 males).  Old **with subjective cognitive decline** (70.6 years; *N* = 16, 4 males). | Demonstrates associations between sensory, motor, and cognitive functioning and contributes to characterize subjective cognitive decline. | No  Yes  Yes |
| Carr et al., (2020) | *StreetLab*: VR street scene, curved projection screen combined with surround sound loudspeakers, and strain gage force plate. | Healthy young (25.4 years; *N* = 16, 9 males).  Healthy old (66.4 years, *N* = 14, 5 males). | Age-related declines in postural control and auditory processing. Inreased listening demands resulting in poorer balance, particularly in older adults. | No  Yes  Yes |
| Chen et al., (2020) | *Kinect 2.0*-incorporated system to capture and generate 3D models of the elderly and immerse them in an interactive virtual environment through screen projection. | Healthy old (71.5 years, *N* = 25, 16 males). | Development of AR-based exergame to reduce fall risk.  ***Showed a good user experience***. | Yes  Yes  No |
| De Sanctis et al., (2020) | Visual Go/No-Go response inhibition task while sitting, Go/No-Go task while walking on a treadmill, simultanously recording foot force sensors and EEG. | Healthy controls (34.6 years, *N* = 15, 6 males).  **Multiple sclerosis patients** (34.9 years, *N* = 13, 3 males). | Multiple scleroris group showed dual-task costs when walking, whereas healthy controls showed a dual-task benefit.  Whereas the healthy controls showed modulation of the brain response as a function of task load, this was not the case in the multiple sclerosis group. | No  Yes  Yes |
| Downer et al., (2016) | Walking with concurrent working memory task in treadmill experiment and similar imagined virtual tasks. | Healthy (45.7 years, *N* = 10, 2 males).  **Multiple sclerosis patients** (43.9 years, *N* = 13, 4 males). | No baseline differences in cognition or walking between groups.  Multiple sclerosis participants demonstrated a decrease in number of correct answers during dual-task. | No  Yes  Yes |
| Ferreira-Brito et al., (2020) | *NeuroVRehab.PT*: Image-based fully navigable and interactive virtual supermarket with navigation of medium-sized supermarket, and the use of shopping lists to memorize. | Healthy old (70.92 years, *N* = 110, 28 males). | Proof of concept: Support that *NeuroVRehab.PT* is an ecologically valid platform with clinical applicability in neuro rehabilitation of vascular mild cognitive impaired older persons.  ***Health professionals (N = 7) assessed the platform's rehabilitation potential, clinical applicability, and user experience***. | Yes  Yes  No |
| Frade et al., (2019) | *GestureMaps*: Stationary walking with VR, *Kinect* application, while measuring physiological parameters and pedometer. | **COPD patients** (50-80 years, *N* = 50, both genders). | ***Application proved to be reproducible and valid for evaluating the functional capacity of subjects with COPD***. | No  Yes  Yes |
| Helbing et al., (2020) | *Virtual Indoor Scenes*: Eye tracking VR head-mounted display and headphones, with 100° field of view, screen with sensors: 37 infrared sensors, an accelerometer, and a gyroscope. Immersive, navigable, and realistic virtual environments to investigate memory performance. | Healthy young (24.3 years, *N* = 21, 7 males). | Enables to investigate the most natural processes for encoding and maintaining information, cognition under ecologically valid conditions, critical for understanding adaptive behavior. | No  Yes  Yes |
| Held et al., (2018) | *REWIRE-system*: telerehabilitation for balance and gait training | **First-time stroke patients** with a mild to moderate residual deficit of lower extremities (56 years, *N* = 16, 9 males) | Patients complied to the system and performed on average 71% of the scheduled session, with 99 minutes of training per week.  System is considered autonomous, safe, feasible and can enable intensive rehabilitative therapy at home.  ***Technology Acceptance Model questionnaire showed excellent values after the training.***  ***Patients were satisfied with and motivated in using the system.*** | Yes  No  No |
| Hsieh et al., (2020) | Virtual reality projections in front of treadmill training versus only treadmill training. | **Multiple sclerosis patients** (*N* = 144), six week intervention, 18 sessions, 3/week. | Recording of dual-task gait speed and information processing speed, prior and post training. | No  Yes  Yes |
| Ijaz et al., (2019) | *VR-CogAssess*: While sitting and using joystick, 3D street panoramas through head-mounted device, while smart watch reads heart rate variability and galvanic skin response. | Healthy old (73.2 years; *N* = 42). | Immersive VR is feasible and compatible with standard personal computer for spatial navigation memory assessment. | Yes  Yes  No |
| Imaoka et al., (2020) | Gait analysis on walkway, body balance with stabilometer while wearing head-mounted display for VR. | Healthy young (24.9 years; *N* = 16, 7 males).  Healthy old (73.1 years, *N* = 14, 6 males). | VR environment can trigger body sway in an expected direction, possibility to enhance the sensitivity of balance assessment by integrating immersive VR environments | No  Yes  Yes |
| Janouch et al., (2018) | *Virtual Street Crossing*: Non-motorized treadmill with 195° of horizontal flat screen view, and headphones. | Healthy young (23.2 years; *N* = 63, 23 males).  Healthy old (70.0 years, *N* = 61, 39 males). | Confirmation of age-related decline in multitasking performance, but requires to test future ecologically valid scenarios. | No  Yes  Yes |
| Jiang et al., (2021) | *Bluegrass working memory platform*: Working memory performance and EEG and memory performance while sitting in front of screen. | Healthy old with normal cognition (74.6 years, *N* = 12, 4 males).  Old **with incident mild cognitive impairment** (76.9 years, *N* = 7, 2 males).  Old **with baseline mild cognitive impairment** (76.4 years, *N* = 14, 10 males). | Memory-related neuromarkers detect brain signatures of mild cogntive impairment about five years before diagnosis. | No  Yes  Yes |
| Kafri et al., (2021) | *Virtual Shopping Mall*: Walking on interactive treadmill while projections on wall-mounted screen through *Computer Assisted Rehabilitation Environment Integrated Reality System (CAREN)* with anatomical landmarks recorded through infra-red cameras. Multiple errands test in a real-world mall or the virtual environment. | Healthy young (26.7 years; *N* = 17, 6 males).  Healthy old (71.2 years, *N* = 17, 18 males). | Shows strengths of using virtual environments in assessing cognitive aspects in every-day-life activities | No  Yes  Yes |
| Kizony et al., (2017) | *Virtual Shopping Mall*: Walking on interactive treadmill while projections on wall-mounted screen through *Computer Assisted Rehabilitation Environment Integrated Reality System (CAREN)* with anatomical landmarks recorded through infra-red cameras. Multiple errands test in the virtual environment. | Healthy young (25.6 years; *N* = 10, 4 males).  Healthy old (69.9 years, *N* = 7, 7 males). | No differences between groups in gait parameters. Both groups walked slower in the mall simulation.  The simulation provided a paradigm to assess the interplay between motor and cognitive aspects involved in the efficient performance of a complex task. | No  Yes  Yes |
| LeGear et al., (2016) | Comparing of *Nintendo Wii* intervention and traditional treadmill intervention. Measurements of energy expenditure, heart rate and oxygen saturation, breathlessness, blood pressure. | **COPD patients** (65.0 years, *N* = 10, 5 males). | Gaming technology can provide an exercise program that has similar cardiovascular demand to traditional pulmonary rehabilitation programs for patients with COPD. | No  Yes  No |
| Liao et al., (2019) | *Kinect* system to capture the limb motions and create a full-body 3D virtual map, wearing VR glasses with a motor controller in hands. Virtual reality-based physical and cognitive training: cognitive control, gait performance, dual-task. | Old **with mild cognitive impairment** (74 years; *N* = 34, 11 males). | VR-based physical and cognitive training program of 12 weeks leads to significant improvements in dual-task gait performance in older adults with mild cognitive impairment | No  Yes  Yes |
| Liu et al., (2016) | *Gait Real-time Analysis Interactive Lab*: Treadmill with 3D motion analysis system with a dual-belt, instrumented treadmill and a VR 180° projection screen. Compare overground walking and VR walking. | Healthy (61.6 years, *N* = 48, 22 males).  **COPD patients** (61.9 years, *N* = 61, 38 males). | ***Promising system to assess the walking in patients with COPD and healthy elderly, with good validity and reproducibility***. | No  Yes  Yes |
| Liu et al., (2017) | *GAITRite system*: Comparison walkway gait parameters with pressure-sensitive sensors of conventional physical therapy, cognitive dual-task gait training, or motor dual-task gait training | **Post-stroke patients** (50.2 years, *N* = 28, 24 males) | Cognitive dual-task gait training improved cognitive dual-task gait performance.  Motor dual-task gait training improved motor dual-task gait performance without group significance.  Different types of dual-task gait training should be adopted to enhance different dual-task gait performance in stroke. | No  Yes  Yes |
| Liston et al., (2021) | *HOLOBalance*: body-worn sensors (pressure detecting insoles, intertial measurement unit and a heart rate monitor) a head mounted augmented reality display and a depth camera. | Old **at risk for falls** (*N* = 120), randomised to receive an 8-week home exercise programme. | ***Proof of concept study: (1) determine the safety, acceptability and feasibility*** of *HOLOBalance*  (2) provide data to support sample size estimates for a future trial. | Yes  Yes  No |
| Penko et al., (2018) | *Computerized Analysis Rehabilitation ENvironment:* extended virtual reality 10-camera 3D motion capture system, 180° curved projection screen and a six degree of freedom motion platform with a treadmill using 31 retroreflective markers. Single and dual-task conditions and biomechanical data capture. | **Parkinson's disease patients** (63.7 years, *N* = 23, 11 males). | Diminished gait performance under dual-task conditions across different cognitive function domains suggests a global Parkinson’s disease-related deficit in information processing and regulation of gait. | No  Yes  Yes |
| Pieruccini-Faria et al., (2021) | Dual-task gait and cognitive performance assessments | Older adults (*N* = 500) **with subjective cognitive impairment, Parkinson disease (PD), mild cognitive impairment (MCI), PD-MCI, Alzheimer’s disease (AD), PD dementia,**  **Lewy body dementia, and frontotemporal dementia**, as well cognitive normal controls. | Of 11 quantitative gait parameters, four independent gait domains were identified: rhythm, pace, variability, and postural control, that enabled group comparisons and classification analysis.  Gait variability was associated with lower cognitive performance and accurately discriminated AD from other neurodegenerative and cognitive conditions. | No  Yes  Yes |
| Solis-Escalante et al., (2019) | *Radboud Falls Simulator*: Dynamic posturography system for investigating standing balance, movable platform in front of computer screen and high-density-EEG. | Healthy young (24.2 years; *N* = 10, 6 males). | Insights into the motor cortical dynamics during the control of human balance. | No  Yes  Yes |
| Souza-Silva et al., (2019) | *Virtual Subway*: Seated and viewing a virtual environment displayed in a helmet mounted display with a 60˚ diagonal field of view. Multi-tasking: phone messaging and the perception of obstacles. | Healthy young (24 years; *N* = 18, 9 males).  Healthy old (68 years, *N* = 15, 7 males). | Text messages prolong the detection of approaching pedestrians, compromising safe ambulation in community environments.  Older adults, may be at even greater risk of collision. | No  Yes  Yes |
| Sutanto et al., (2019) | *Wii Fit system*: Balance board with interactive games. | Healthy (65.6 years, *N* = 10, 10 males).  **COPD patients** (65.1 years, *N* = 9, 8 males). | Both groups experienced significant improvements, without any benefit to a well conducted standard exercise training program in COPD patients | No  Yes  Yes |
| Tarnanas et al., (2013) | *Fire Evacuation Virtual Reality Day-Out Task*: VR reality with activities of daily living, leap motion sensor integrated treadmill, *Kinect* sensor and curved projection screen. | Healthy old normal cognition (72.6 years; *N* = 72, 25 males).  Old **with amnestic-type mild cognitive impairment** (72.8 years; *N* = 65, 30 males).  Old **with mild Alzheimer-type dementia** (72.6 years; *N* = 68, 33 males). | Virtual reality measures of functional ability seem more sensitive to functional impairment than qualitative measures in predementia, thus accurately differentiating from healthy controls. | No  Yes  Yes |
| Van Beek et al., (2019) | *Leap Motion Controller*: Dexterity intervention with laptop, markerless motion sensing system that tracks the motion of both forearms, wrists, and hands. | **Parkinson's disease patients** (65.4 years, *N* = 10, 3 males). | Participants with impaired dexterity significantly improved.  ***High adherence and increased motivation.***  ***Usability of system was acceptable to very good.*** | Yes  Yes  No |
| Wechsler et al., (2018) | *Immersive Driving Simulator*: Car seat in front of three screens, with a total viewing angle of 195°, a headset with microphone | Healthy young (23.2 years; *N* = 63, 23 males).  Healthy old (70.0 years, *N* = 61, 39 males). | Multitasking deteriorates in older age not only in typical laboratory paradigms, but also in paradigms that require orchestration of dual-tasking and task switching. | No  Yes  Yes |
